# New York County

**Published:** 1898-05

**Authors:** Robert W. Ellis

**Affiliations:** Secretary


Secretary.

### VETERINARY MEDICAL ASSOCIATION OF NEW YORK COUNTY.

THE regular monthly meeting of the Association was called to order at the Academy of Medicine on March 2, 8.45 P.M., the President, Dr. Huidekoper, in the chair. The following members responded to roll-call: Drs. Ackerman, Burns, Bretherton, Bell, C. C. Cattanach, J. S. Cattanach, J. S. Cattanach, Jr., Delaney, Dair, Ellis, Foy, Gill, Grenside, Huidekoper, Hanson, MacKellar, Neher, O'Shea, and Ryder. The minutes of the previous meeting were read and approved.

Reports of Committees: Board of Censors, H. D. Gill, chairman, reported no business in hand. Judiciary, Dr. Ryder, as chairman, *pro tem.*, reported in Dr. O'Shea's absence. Committee on Ways and Means, Dr. Bell, chairman, reported progress. Moved and second that the reports of the various committees be accepted; carried.

Reading of papers: Dr. Gill took the initiative by reading a paper on "The Relation of the Veterinarian to the Board of Health as Meat- and Milk-inspectors."[1] Discussion followed by Dr. Austin Peters, of Boston; Dr. W. Horace Hoskins, of Philadelphia; Dr. Leonard Pearson, Philadelphia; Dr. R. W. Hickman, U. S. B. A. I., of New York, and Dr. W. Herbert Lowe, of Paterson, N. J., State Veterinarian of New Jersey and late Veterinary Inspector to the Port of New York. The discussion was closed by the essayist.

Under the head of new business it was moved and seconded, that a committee of three be appointed to draw up resolutions on the death of Theodore Birdsall; carried. The following resolution was offered by Dr. Bell:

*Resolved*, That a committee be appointed to draw up resolutions expressive of the feelings of this meeting in relation to the importance of meat- and milk-inspection to the health of the general public, and the necessity for the appointment of veterinarians by boards of health as such inspectors.

Moved by Dr. O'Shea, that a vote of thanks be extended to Dr. Gill for his paper, and to Drs. Peters, Hoskins, Pearson, Hickman, and Lowe for the discussion. Seconded; carried.

Moved and seconded that the meeting adjourn; carried.

THE regular monthly meeting was called to order in Room 37, New York Academy of Medicine, at 8.30 P.M., April 6th, Dr. Huidekoper presiding. On roll-call the following members responded: Drs. Bretherton, C. C. Cattanach, J. S. Cattanach, J. S. Cattanach, Jr., Dickson, Delaney, Dair, Ellis, Farley, Gill, Huidekoper, Lamkin, Machan, MacKellar, Murphy, Neher, O'Shea, and Ryder (18). As visitors: Drs. L. Nicolas, Charles Hall, Olof Schwarzkopf, C. E. Clayton, M. Kenny, J. F. DeVine, J. William Fink, E. F. Sanford, Charles S. Atchison, James W. Walker, B. Günther, August D. Moeller, George P. Biggs, M.D., A. W. Clement.

Report of Judiciary Committee: Dr. O'Shea, Chairman, reported that

[1] See page 295.

the bill introduced to allow Charles McCormick, of the City and County of Albany, to practise, although it had passed the Assembly, was killed in the Senate, and that the bill exempting veterinarians from jury-duty in New York and Kings Counties had passed both Houses and was in the hands of the Governor, awaiting his signature to become a law. Moved and seconded, that the report be accepted as read; carried.

Ways and Means Committee: Dr. Ryder, Chairman, *pro tem.*, reported for this committee, that at the May meeting Dr. J. S. Cattanach will read a paper on "Economy in the Practice of Veterinary Medicine," and that Dr. J. S. Lamkin will read a paper on "Parturient Apoplexy." Moved and seconded that the report be accepted and placed on file; carried.

Testimonial Committee: In behalf of Dr. O'Shea, Dr. J. S. Cattanach, Chairman, reported that he had done considerable work, but required more men on the committee, and requested that two more be appointed. This request was granted, and the President appointed to act with that committee Drs. Delaney and Grenside.

Papers: Dr. Huidekoper delivered a most interesting and instructive discourse on "Navicular Disease." The discussion which followed was led by Dr. A. W. Clement, of Baltimore, who mentioned as a treatment for this condition what he termed "Surface Firing" with the thermo-cautery. This treatment, in which the skin is *not* punctured, is repeated daily for a time, then every second day, with good results. Drs. Neher, Schwarzkopf, and Clayton participated in the discussion that followed. Moved and seconded that the discussion close; carried.

Moved and seconded that a vote of thanks be extended to the essayist for his most excellent address; carried.

Dr. Ryder next read a paper entitled "College and State Examinations." Dr. Biggs led in the discussion of this paper, and was followed by Dr. A. W. Clement, member of the State Board of Examiners of Maryland. Among those who discussed this important subject were Drs. Gill, Moeller, DeVine, and Huidekoper. Discussion was closed by Dr. Ryder. Moved and seconded that a vote of thanks be extended to Dr. Ryder; carried.

A communication from Dr. Roscoe R. Bell, tendering his resignation as Chairman of the Committee on Ways and Means of the Society, was read by the Secretary. Moved and seconded that this resignation be referred to the Board of Censors to report on at the next meeting; carried.

Moved and seconded that the meeting adjourn; carried.

ROBERT W. ELLIS, D.V.S.,
Secretary.

